# Eyelash Impalement of Iris After Uncomplicated Cataract Surgery

**DOI:** 10.1155/crop/6657874

**Published:** 2025-04-03

**Authors:** Jenny Y. Gan, Michael M. Lin

**Affiliations:** ^1^Harvard Medical School, Boston, Massachusetts, USA; ^2^Department of Ophthalmology, Massachusetts Eye and Ear, Boston, Massachusetts, USA

**Keywords:** cataract surgery, intraocular cilia, intraocular eyelash, intraocular foreign body, phacoemulsification

## Abstract

This report highlights the first known report of an intraocular eyelash embedded in the inferior midperipheral iris following a routine cataract surgery. A 74-year-old female presented 5 months postoperatively from a full-thickness macular hole repair with a 3+ nuclear sclerotic cataract. She underwent an uneventful left eye cataract extraction with insertion of posterior chamber intraocular lens (PCIOL). Review of surgical video revealed well-draped eyelashes and no introduction of an eyelash into the anterior chamber at any point during surgery. The first postoperative visit was notable for no foreign bodies and centered PCIOL. Then, 1 week postoperatively, the anterior segment examination revealed a linear foreign body embedded in the inferior midperipheral iris, without corneal endothelium touch. The foreign body was removed in the operating room and revealed to be an eyelash by pathology. The patient's visual acuity during her first two postoperative visits was 20/30 and improved to 20/20 with refraction at her 1-month postoperative visit. There was minimal postoperative inflammation and no evidence of endophthalmitis. To prevent introduction of an intraocular eyelash associated with cataract surgery, appropriate measures intraoperatively and postoperatively should be taken, including meticulous draping of the eyelashes and lids, diligent corneal wound hydration, and clear patient education.

**Summary**

• Intraocular eyelashes have been reported after ophthalmic surgery in rare instances.
• After a literature review, we did not find any prior report of an eyelash embedded in the iris after an uncomplicated cataract surgery.
• Avoidance of eye rubbing in the early postoperative period is essential for proper healing.

## 1. Introduction

Cataracts are the leading cause of blindness in the world, and cataract surgery is one of the most common intraocular surgeries performed worldwide, with an increasing incidence of cataract surgeries over time [1–3]. While complications from cataract surgery are rare, the most common intraoperative complication is posterior capsular rupture (PCR), and the most common postoperative complications include posterior capsular opacification, retinal detachment, and endophthalmitis [4, 5]. Rarer complications include those involving an intraocular eyelash, with case reports reporting eyelashes in the corneal incision and the anterior chamber. An even less common complication is a single case report of an eyelash embedded in the iris after an anterior chamber intraocular lens, which was discovered after 16 years [6]. After conducting a literature review on intraocular eyelashes utilizing PubMed using the key words “intraocular eyelash” or “intraocular cilia” and “cataract surgery” or “phacoemulsification,” we did not find any prior reports of an eyelash embedded in the iris after an uncomplicated cataract surgery with insertion of a posterior chamber intraocular lens (PCIOL) in the capsular bag, which we describe in this case report.

## 2. Case Report

A 74-year-old female presented for follow-up of her cataracts and glaucoma suspect status due to increased cup to disc ratios in both eyes. Her past ocular history was notable for full thickness macular hole of her left eye that had been treated with pars plana vitrectomy, membrane peeling, fluid air exchange, and 20% SF6 gas tamponade. Prior to this surgery, BCVA of her left eye had been 20/200, and it improved to 20/40 at the 5-week postoperative visit. By 5 months after surgery, BCVA had worsened to 20/70. The anterior segment showed a 3+ nuclear sclerotic cataract with an opalescent core and fine fleck opacities. The patient then underwent an uneventful left eye cataract extraction with an inferotemporal paracentesis incision and a superotemporal main clear corneal incision, insertion of PCIOL, and intracameral moxifloxacin. At the first postoperative visit, the patient's anterior segment examination was notable for no foreign bodies and a centered PCIOL. Additionally, both temporal incisions were determined to be watertight and Seidel negative.

**Figure 1:**
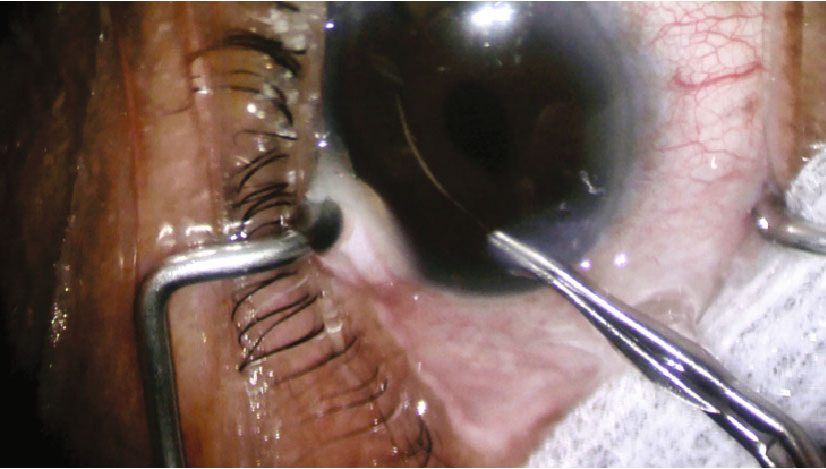
A 74-year-old female underwent uneventful left eye cataract extraction with insertion of a posterior chamber intraocular lens for a 3+ nuclear sclerotic cataract. At her 1-week postoperative visit, a linear foreign body was discovered embedded in her inferior midperipheral iris. The foreign body was fixed to the iris and immobile. The patient returned to the operating room, where the foreign body was removed and confirmed to be an eyelash by pathology.

At the patient's 1-week postoperative visit, a linear foreign body was discovered embedded in her inferior midperipheral iris, without corneal endothelium touch. At this time, her BCVA was 20/40 and IOP was 16. There was less than 1+ cell in the anterior chamber and no hypopyon or conjunctival injection. The patient returned to the operating room, where the foreign body was removed with the aid of viscoelastic and angled tying forceps (Figure 1). Intracameral moxifloxacin was injected at the conclusion of surgery again. The foreign body was later confirmed to be an eyelash by pathology. The patient's BCVA was her first two postoperative visits was 20/30 and improved to 20/20 with refraction at her 1-month postoperative visit. IOP remained stable between 12 and 21 mmHg without treatment, and there was minimal postoperative inflammation and no evidence of endophthalmitis.

## 3. Discussion

While complications involving intraocular eyelashes during cataract surgery are relatively rare, there have been nine previous case reports of this type of complication. Of these case reports, three report an eyelash in the clear corneal incision [7–9], and four report the eyelash in the anterior chamber [10–13], with 1 noting the eyelash to migrate from the anterior chamber to the peripheral iris through the posterior chamber and pupil before coming to rest in the inferior angle [12]. The remaining 2 reported cases describe a case of an intraocular eyelash posterior to the PCIOL but anterior to the posterior capsule [14] and a case of an eyelash embedded in the iris following an anterior chamber IOL [6]. In only one of these nine case reports was endophthalmitis reported [13].

In addition to intraocular eyelashes, there have been reports of other types of foreign bodies discovered after routine cataract surgery; the composition of these foreign bodies largely includes metal, organic fiber, and plastic. Retained metal foreign bodies are theorized to be most likely from surgical instruments, such as the phacoemulsification tip, a surgical needle, or an aspiration cannula [15–18]. In most cases, the metallic foreign body was discovered on a postoperative examination and typically found to be in the iris, though the particles were also found to be in the anterior chamber and the corneal stroma [16, 18–21]. Some patients were asymptomatic, while others presented with decreased visual acuity and cystoid macular edema [17–20, 22, 23]. Other case reports note foreign bodies mostly made of organic fibers that were found on postoperative visits via slit lamp examination in the iris and anterior chamber [24, 25]. The cause of these fibers was theorized to be from the cotton sterile drapes, cotton balls, and surgical gauze [26–28]. Unlike the metal retained objects, the cotton fibers were generally asymptomatic, and studies detected no significant effect between removing the fiber versus clinical monitoring [24, 28]. Finally, the last category of foreign retained objects includes plastic particles which presented in patients with decreased visual acuity, corneal edema, and bullous keratopathy and which were found to be attached to the iris or to the posterior surface of the IOL [29–32]. The authors of the case reports suggested that the plastic particles originated from the packaging of the IOL, the cartridge of the IOL, or the suture packaging [30–33].

In this case, the patient did not report any vigorous eye rubbing or related trauma that could have caused introduction of an eyelash into the anterior chamber. Careful review of the video of the initial cataract surgery revealed well-draped eyelashes and no introduction of an eyelash into the anterior chamber at any point during surgery. Because the examination at the conclusion of the initial operation did not reveal the intraocular eyelash, the foreign body was thought to have been introduced in the first week following the surgery through the corneal incision. An alternative possibility is that the eyelash was introduced into the eye during pars plana vitrectomy and membrane peeling that had occurred 5 months prior to cataract surgery, and the fluid shifts during and after cataract surgery allowed the retained, previously undetected intraocular eyelash to reveal itself. Yet another hypothesis for the cause of this complication is that it was due to the location of the incision temporally, which could increase the risk of an embedded eyelash, as the upper eyelid does not cover the incision. This cause was also previously hypothesized by Cheong and Wee [8].

This case is unique because it is the first report in the literature of an intraocular eyelash embedded in the inferior midperipheral iris following a routine phacoemulsification with no intraoperative complications. The position of the eyelash allowed for an uneventful removal, and it was fortunate that the patient did not experience any other complications such as endophthalmitis, with her BCVA and IOP remaining stable.

## 4. Conclusions

To prevent similar complications such as this case of an intraocular eyelash detected after cataract surgery, appropriate measures intraoperatively and postoperatively should be taken. These include meticulous draping of the eyelashes and lids, as well as diligent cornea wound hydration to ensure the incisions are watertight. Additionally, clear patient education to avoid eye rubbing following surgery is important for prevention.

## Data Availability

The data that support the findings of this study are available from the corresponding author upon reasonable request.
